# Increasing similarities between young adults’ smoking and snus use in Norway: a study of the trends and stages of smoking and snus epidemic from 2010 to 2018

**DOI:** 10.1186/s12889-020-09604-6

**Published:** 2020-10-06

**Authors:** Tore Tjora, Jens Christoffer Skogen, Børge Sivertsen

**Affiliations:** 1 Department of Social Studies, Faculty of Social Sciences, University of Stavanger, Stavanger, Norway; 2 Department of Health Promotion, Norwegian Institute of Public Health, Bergen, Norway; 3 Alcohol & Drug Research Western Norway, Stavanger University Hospital, Stavanger, Norway; 4 Department of Public Health, Faculty of Health Sciences, University of Stavanger, Stavanger, Norway; 5 Department of Research & Innovation, Helse Fonna HF, Haugesund, Norway; 6 Department of Mental Health, Norwegian University of Science and Technology, Trondheim, Norway

**Keywords:** “Snus use”, “Smoking”, “Socioeconomic status”, “Trends”, “Stages”, “SES”

## Abstract

**Background:**

The prevalence of smoking has been decreasing in Norway for decades. In contrast, the prevalence of snus use has recently increased substantially, especially among females. While there is a clear social gradient in smoking, with a higher smoking prevalence among individuals with low socioeconomic status (SES), a possible social gradient in snus use has been less studied. The aim of the current study was to investigate the trends of smoking and snus use and to examine whether ongoing changes in snus use are similar to prior smoking epidemic development.

**Methods:**

The study was based on the 2010 (*n* = 5836), 2014 (*n* = 13,319) and 2018 (*n* = 24,515) waves from a nation-wide, cross-sectional, health survey of higher education in Norway (the SHoT study). Variables on smoking, snus use, gender, age and SES were used. Chi-square tests and logistic regression analyses were used to test significance, and Mantel–Haenszel weights were used to test the trends in stratified cross-tabulations.

**Results:**

Daily smoking decreased from 5.9 to 1.5% between 2010 and 2018, while daily snus use increased from 13.4 to 19.9%. Female snus use almost doubled, from 10.9 to 19.2%. Low SES was associated with both daily smoking and snus use across all three waves. Occasional smoking was also associated with low SES at all waves, but occasional snus use was only associated with low SES in 2010. There were no significant changes over time in either the association between occasional or daily smoking and SES or the association between occasional or daily snus use and SES.

**Conclusions:**

The overall smoking decrease indicated that the Norwegian smoking epidemic is in its latest stage. Steady male snus use, doubled female snus use and a clear social gradient in snus use all indicate that the snus epidemic in Norway has progressed. If this trend continues, a main implication is that snus prevalence will soon peak, first in males and then in females.

## Background

Although the percentage of people smoking cigarettes has declined in recent years, due to population growth, the total number of smokers has actually increased [1] to nearly 1 billion people worldwide. Smoking is still the second leading risk factor for premature mortality and disability [2], claiming more than 5 million lives yearly since 1990 [1].

Lopez and colleagues’ descriptive model of the cigarette smoking epidemic is central to our understanding of smoking epidemics in economically developed countries [3, 4]. The model divides the cigarette epidemic into four stages. The first two stages are characterized by smoking prevalence increasing in both genders, first in males (stage I) and later in females (stage II). Stage III is special, as male smoking prevalence decreases, while female smoking continues to increase. Stage IV is characterized by decreasing smoking prevalence in both males and females [3].

There may be several reasons to the twenty years male head-start described in Lopez’ first stage, including gender difference in social norms [5] and a shift from marketing focusing on male smoking to focus on both [5]. In the US and UK, the relative small gender gap of twenty years is attributed to changes in norms as a result of World War II [4]. In Norway, tobacco marketing has been banned since the seventies [6], which may partly explain both the low prevalence and diminished gender differences. A more recent update of the Lopez model shows that the overall model still describes the smoking epidemic accurately, but despite similar trends, indicates that the female smoking development currently lags behind that of males [4].

Similar to many economically developed countries, following the model postulated by Lopez et al., the smoking prevalence in Norway has also decreased in both adult [7] and adolescent [8] populations. Parallel to the general decrease in smoking, there has been a clear social diffusion process (diffusion is the process of how innovations, trends, etc. spread through social systems over time [9]). The smoking prevalence in developed countries is highest in lower socioeconomic status (SES) groups [10], which is suggested to be a result of both higher smoking initiation and less smoking cessation in lower-SES groups [10, 11].

Decades ago, in the first stages of the smoking epidemic, the trend of smoking initiation spread through social networks [12]. Now, the trend of smoking cessation seems to be spreading in a similar manner [13], meaning that the “early adopters” have taken on the cessation trend first, as they did the smoking trend decades ago, further reinforcing the widely documented social gradient in smoking [10]. Such an emerging social gradient, the stabilization of male smoking and increased female smoking are three hallmarks of the transition from stage II to stage III according to Lopez [3]. The combination of great health hazards and a clear social gradient makes smoking a significant contributor to the general social gradient observed across health outcomes [14].

In contrast to smoking, which strongly affected the disease pattern in the US during the twentieth century [15], snus use is relatively new, at least outside of Scandinavia. Snus, a form of moist smokeless tobacco used orally, has been used in Scandinavia since the early 1900s [16, 17]. In EU countries, except Sweden, snus has been banned since 1992 [18]. Despite this selling ban, snus use in Finland is increasing rapidly [19]. Outside of Europe, while banned in, e.g., Australia [20], snus is not banned in the US. Despite different forms of smokeless tobacco products being available locally in the US for many years, snus was first introduced in 2010 [21]. In Canada, snus is not banned, but a prevalence of smokeless tobacco in general of approximately 1% for adults in both 2013 and 2015 [22] indicates a very low prevalence of snus use. In Norway, the official statistics on snus use have been collected since 2008, whereas comparative trend data on smoking have existed since the 1970s.

A recent review concluded that snus use is associated with health hazards [23], although not to the same extent as smoking. Another potential negative consequence of snus use is the possibility that it functions as a gateway to the later onset of smoking. Although a switch from smoking to snus use is considered beneficial, as it reduces harm [18, 24], there have also been studies suggesting that the groups at risk of the initiation of smoking and snus use are overlapping [8]. Such findings lend support to a snus-to-smoking gateway theory. However, findings are mixed, and a Swedish study did support snus being a gateway to smoking [25].

In Norway, the observed decrease in smoking has occurred alongside the increased use of snus [7], a trend that is particularly pronounced among adolescents and females [8, 26]. In contrast to smoking, the social gradient in snus use has been less clear in adults [27]. A Swedish study on adults in 2010 concluded that snus use was associated with low SES in men but with high SES in women [27]. A recent study from Finland showed a similar social gradient in both smoking and snus use [19]. In contrast, a Norwegian study of adolescents from 2010 concluded that there was no social gradient in snus use, which is in sharp contrast to the case of smoking in this age cohort [26]. A recent report shows that low SES was associated with adolescent snus use [7]. The present study is based on young adults, when any associations between both snus use and SES and smoking and SES are established.

The first aim of this study was to investigate the trends in tobacco use, both smoking and snus use, from 2010 to 2018 in Norwegian young adults. The second aim was to investigate whether the ongoing change in snus use in Norway may be similar to the changes described in the transition between stage II and stage III in Lopez and colleagues’ smoking epidemic model [3, 4]. Such a finding will lend support to the theory postulating a snus use epidemic following the same stages as the smoking epidemic, further postulating an oncoming peak and subsequent decrease in snus use, first for males and then for females.

## Methods

The SHoT study (Students’ Health and Wellbeing Study) is a national student survey for higher education in Norway, initiated by the three largest welfare associations. To date, three surveys of the student population (aged 18–35 years) in Norway have been completed (2010, 2014 and 2018), and all three waves were collected electronically through a web-based platform. The three studies were conducted separately (not a longitudinal data collection). The details of the SHoT study have been published elsewhere [28, 29]. The SHoT2018 was conducted between 6 February and 5 April 2018, inviting by email all full-time Norwegian students pursuing higher education (both in Norway and abroad) to participate. For the SHoT2018 study, 162,512 students fulfilled the inclusion criteria, of whom 50,054 students completed the online questionnaires, yielding a response rate of 30.8%. The SHoT2014 study was conducted between 24 February and 27 March 2014. An invitation email containing a link to an anonymous online questionnaire was sent to 47,514 randomly selected students and stratified by study institutions, faculties and departments. The overall response rate was 28.5% and included 13,525 students. The SHoT2010 study was conducted between 11 October and 8 November 2010. The target group was a random sample of 26,779 Norwegian full-time students, of whom 6053 students completed the survey, yielding a response rate of 22.6%.

### Instruments

Smoking was measured with one question: “Do you smoke?” The response options included “Yes, daily”, “Yes, occasionally” and “No”. A dichotomous variable, “daily smoking”, was operationalized as smoking daily compared to occasionally smoking or not smoking.

Snus use was measured with one question: “Do you use snus or something similar?” The response options included “Yes, daily”, “Yes, occasionally” and “No”. A dichotomous variable, “daily snus user”, was operationalized as using snus daily compared to occasionally snus use or no snus use.

A variable on tobacco use was constructed by combining the abovementioned snus use and smoking questions, making a tobacco use variable ranging from no tobacco use to occasional tobacco use to daily tobacco use.

All participants indicated their gender and age, and age was in the questionnaire grouped into four groups: from 18 to 20 years, from 21 to 22 years, from 23 to 25 years and from 26 to 34 years. A dichotomous variable “younger” was defined as being in the 18- to 20-year group compared to all age groups. “Older” was defined as being in the 26- to 34-year group, compared to all other age groups.

SES was measured with one question: “Has it during the last 12 months happened that you or your household has had difficulties coping with household spending, such as food, transport, housing?” The response options included “Never”, “Rarely”, “Occasionally” and “Often”. A dichotomous variable, “low SES”, was defined as answering “often” on the SES question compared to all other responses. “High SES” was defined as answering “never”, in comparisons to all other responses.

### Statistical analysis

Statistical analyses were computed using Stata/IC 15.1. First, we performed a two-way analysis of variance (ANOVA), examining how key variables were distributed across smoking and snus use in 2010, 2014 and 2018 (Table 1). We tested for differences using chi-square tests. Second, we examined the trends in the associations between single predictors and both daily smoking and daily snus use by running multiple case-control logistic regression analyses stratified by year (Table 2). Third, we examined the associations between low SES and both smoking and snus use in every wave while adjusting for gender and age (Table 3).

**Table 1:** The distribution of smoking and snus use across key predictors from 2010 to 2018

**Tobacco**												
			Daily	Occ^a^	No	n	diff^b^					
Overall \tobacco use from 2010 to 2018		2010	18.8%	14.1%	67.2%	5836	χ^2^ = 79.29, df = 4, *p* < 0.001					
		2014	20.2%	10.2%	69.6%	13,319						
		2018	21.2%	12.1%	66.8%	24,515						
**Smoking**								**Snus**				
			Daily	Occ^a^	No	n	diff^b^	Daily	Occ^a^	No	n	diff^b^
Smoking and snus use from 2010 to 2018		2010	5.9%	9.1%	85.0%	5813	χ^2^ = 446.85, df = 4, p < 0.001	13.4%	9.8%	76.9%	5815	χ^2^ = 174.54, df = 4, *p* < 0.001
		2014	2.7%	6.3%	91.0%	13,294		17.9%	7.1%	75.0%	13,311	
		2018	1.5%	8.3%	90.1%	24,104		19.9%	8.4%	71.6%	24,441	
Gender	Male	2010	4.4%	9.0%	86.6%	1995	χ^2^ = 12.92, df = 2, *p* = 0.002	18.2%	9.8%	72.1%	1998	χ^2^ = 61.80, df = 2, p < 0.001
	Female	2010	6.7%	9.2%	84.1%	3818		10.9%	9.8%	79.4%	3817	
Gender	Male	2014	2.3%	8.1%	89.6%	4455	χ^2^ = 43.41, df = 2, p < 0.001	21.4%	6.8%	71.9%	4460	χ^2^ = 55.39, df = 2, *p* < 0.001
	Female	2014	2.9%	5.3%	91.7%	8839		16.1%	7.3%	76.5%	8851	
Gender	Male	2018	2.0%	11.7%	86.4%	6131	χ^2^ = 133.85, df = 2, p < 0.001	22.0%	8.5%	69.5%	6225	χ^2^ = 22.58, df = 2, p < 0.001
	Female	2018	1.4%	7.2%	91.4%	17,973		19.2%	8.4%	72.4%	18,216	
Age, grouped	18–20	2010	3.7%	7.3%	89.0%	1185	χ^2^ = 103.01, df = 6, p < 0.001	11.6%	8.7%	79.7%	1181	χ^2^ = 25.34, df = 6, p < 0.001
	21–22	2010	4.7%	8.7%	86.6%	1644		14.8%	11.4%	73.7%	1645	
	23–25	2010	4.9%	9.4%	85.8%	1852		14.3%	10.0%	75.7%	1852	
	26–34	2010	11.8%	11.1%	77.1%	1132		11.5%	7.9%	80.6%	1137	
Age, grouped	18–20	2014	1.1%	5.3%	93.7%	1709	χ^2^ = 202.16, df = 6, p < 0.001	13.1%	5.8%	81.1%	1712	χ^2^ = 60.56, df = 6, *p* < 0.001
	21–22	2014	1.4%	5.7%	92.9%	3578		16.8%	7.2%	76.1%	3581	
	23–25	2014	2.1%	5.9%	92.0%	4761		20.3%	7.0%	72.7%	4774	
	26–34	2014	6.0%	7.9%	86.2%	3246		18.2%	8.0%	73.8%	3244	
Age, grouped	18–20	2018	0.9%	8.6%	90.6%	4561	χ^2^ = 203.37, df = 6, p < 0.001	14.2%	7.8%	78.0%	4617	χ^2^ = 185.06, df = 6, p < 0.001
	21–22	2018	1.0%	8.3%	90.7%	7703		18.9%	9.0%	72.1%	7801	
	23–25	2018	1.3%	8.3%	90.4%	7774		22.4%	8.8%	68.7%	7911	
	26–34	2018	4.2%	8.0%	87.8%	3849		23.6%	7.1%	69.3%	3892	
Problems paying bill (SES)	Never	2010	2.4%	6.2%	91.4%	2387	χ^2^ = 233.89, df = 6, p < 0.001	9.1%	8.2%	82.7%	2383	χ^2^ = 101.82, df = 6, p < 0.001
	Seldom	2010	5.9%	10.4%	83.6%	1430		14.0%	11.1%	74.9%	1434	
	Occ^a^	2010	7.8%	10.9%	81.3%	1463		17.0%	9.9%	73.1%	1467	
	Often	2010	16.8%	13.8%	69.4%	523		20.7%	12.6%	66.7%	522	
Problems paying bill	Never	2014	1.1%	4.0%	94.8%	4569	χ^2^ = 212.22, df = 6, p < 0.001	11.3%	5.6%	83.1%	4577	χ^2^ = 339.62, df = 6, p < 0.001
	Seldom	2014	2.2%	5.8%	92.0%	3293		17.8%	7.8%	74.4%	3298	
	Occ^a^	2014	3.6%	8.1%	88.3%	3858		21.6%	7.9%	70.4%	3857	
	Often	2014	5.9%	9.3%	84.8%	1554		28.3%	8.3%	63.4%	1559	
Problems paying bill	Never	2018	0.8%	6.4%	92.8%	11,576	χ^2^ = 346.35, df = 6, p < 0.001	13.9%	7.7%	78.4%	11,699	χ^2^ = 724.46, df = 6, p < 0.001
	Seldom	2018	1.4%	8.7%	89.9%	5490		21.0%	9.0%	70.1%	5582	
	Occ^a^	2018	2.4%	10.3%	87.3%	5178		27.6%	9.1%	63.3%	5268	
	Often	2018	4.4%	14.1%	81.5%	1909		32.8%	9.3%	58.0%	1941	

^a^occ = occasionally, ^b^diff = difference

**Table 2:** Trends from 2010 to 2018 of the key variables of smoking and snus use

	Daily smoking				Daily snus use			
	2010	2014	2018	Test of homogeneity 2010 to 2018	2010	2014	2018	Test of homogeneity 2010 to 2018
	OR	OR	OR	Significance	OR	OR	OR	Significance
Male	0.64	0.77	1.42	p < 0.001^a^	1.82	1.41	1.18	p < 0.001^a^
Young	0.55	0.35	0.50	*p* = 0.28	0.82	0.66	0.61	*p* < 0.05^a^
Old	2.81	3.72	3.98	*p* = 0.07	0.81	1.03	1.30	p < 0.001^a^
Low SES	3.95	2.70	3.32	*p* = 0.11	1.81	2.00	2.09	*p* = 0.49
High SES	0.27	0.33	0.37	*p* = 0.23	0.51	0.47	0.47	*p* = 0.60

^a^= Significant change from 2010 to 2018

*OR* odds ratio, *SES* socioeconomic status

**Table 3:** Daily smoking and snus use by low socioeconomic status; crude odds ratios are given and adjusted for gender and age

		Daily smoking			Daily snus use		
		2010	2014	2018	2010	2014	2018
Crude odds ratio	Low SES	3.95	2.70	3.32	1.81	2.00	2.09
Adjusted odds ratio	Low SES	3.65	2.47	2.88	1.98	2.06	2.06
	Male	0.66	0.73	1.37	1.88	1.46	1.20
	Old	2.73	3.65	3.61	0.78	NS^a^	1.23

NS^a^ Not significant, omitted in final model

## Results

The number of young adult students who smoked decreased from 5.9% in 2010 to 1.5% in 2018 (Table 1). Additionally, occasional smoking decreased from 2010 to 2018. The snus use over the same period increased from 13.4 to 19.9% (Table 1). Occasional snus use decreased from 9.8% in 2010 to 8.4% in 2018. Taken together, the decrease in smoking and the increase in snus use resulted in an overall increase in daily tobacco use from 2010 to 2018 (Table 1). However, the overall occasional use of tobacco decreased from 2010 to 2018.

### Gender

As detailed in Table 1, in 2010, more women (6.7%) were daily smokers than men (4.4%). The smoking prevalence decreased in both genders from 2010 to 2018, but in 2018, the gender differences were reversed, with more men than women being daily smokers (2.0% versus 1.4%, respectively). The trend analysis showed similar findings, with male gender being a protective factor regarding daily smoking in 2010 (OR = 0.64, CI = 0.49–0.82) but a risk factor for daily smoking in 2018 (OR = 1.42, CI = 1.13–1.77) (Table 2). Gender was the only key variable associated with significant changes in smoking from 2010 to 2018 (test of homogeneity: χ^2^ = 25.51, df = 2, *p* < 0.01) (Table 2).

Regarding daily snus use, the prevalence was highest among men across all waves, but the prevalence among women nearly doubled from 2010 to 2018 (see Table 1 for details). In 2010, the gender difference was large, with 18.2% of men being daily snus users, compared to 10.9% of women. In 2018, the difference was smaller; 22.0% of men were daily snus users, compared to 19.2% of women. In contrast to smoking, being male was significantly associated with higher odds for snus use in 2010 (OR = 1.82, CI = 1.56–2.13) compared to 2018 (OR = 1.18, CI = 1.10–1.27) (Table 2).

### Age

The prevalence of smoking was highest in the oldest age group across all waves. In 2010, the group of oldest students (26–34 years) had a higher prevalence of daily smoking (11.8%) compared to the other groups (ranging from 3.7 to 4.9%; Table 1). In 2014 and 2018, the tendency was the same, but the differences were smaller; the oldest students had a prevalence of 6.0% in 2014 and 4.2% in 2018, compared to the younger age groups, ranging from 1.1 to 2.1% in 2014 and 0.9 and 1.3% in 2018. There were no significant trends from 2010 to 2018 regarding smoking and age (Table 2).

The age distribution of snus use was different in two regards: first, there were smaller age group differences, and second, the oldest students did not have the highest prevalence in every wave. In 2010, daily snus use was least common in both the youngest and oldest students (18–20 years: 11.6%, 26–34 years: 11.5%). In 2014, the snus use prevalence ranged from 13.1% in the youngest group to 20.3% in the group of students from 23 to 25 years (Table 1). In 2018, daily snus use had the lowest prevalence in the youngest group (14.2%) and the highest prevalence in the group with oldest students (23.6%) (Table 1). Unlike smoking, the association between snus and age changed significantly across the three waves: being young became a protective factor against snus use in 2018 (OR = 0.61, CI = 0.56–0.67) (Table 2). Being in the oldest age group changed from being a protective factor in 2010 (OR = 0.81, CI = 0.66–1.00) to being a risk factor in 2018 (OR = 1.30, CI = 1.19–1.41) (Table 2).

### Socioeconomic status

Not being able to manage household spending, the question used to measure SES, had a very similar dose-response relationship with both daily smoking and daily snus use in all waves. These findings were stable across all waves (Table 2). Individuals never having problems with household spending in 2018 had a smoking prevalence of 0.8%, whereas those reporting often having such financial problems had a smoking prevalence of 4.4%. A similar graded association with financial problems was observed for snus use; the worse the financial problems were, the higher the snus prevalence (see Table 1 for details).

The association between low SES and both daily smoking and daily snus use was still significant in all three waves after adjusting for gender and age (Table 3). Participants with low SES had an approximately three times higher chance of being daily smokers across all three waves (2010: OR = 3.65, 2014: OR = 2.47, 2018: OR = 2.88) and twice the chance of being a daily snus user (2010: OR = 1.98, 2014: OR = 2.06, 2018: OR = 2.06) (Table 3).

## Discussion

The main finding is that due to increased snus use, despite declining smoking prevalence, the prevalence of overall tobacco use increased slightly from 2010 to 2018. Another important finding is that snus users are becoming more similar to smokers from 2010 to 2018 in four important aspects. First, the gender difference in snus use is decreasing, as females take up snus use. Second, low SES is associated with both snus use and smoking. Third, being in the oldest group of young adults became a risk factor in 2018, and being in the oldest group was a risk factor for smoking across all waves. Fourth, being in the youngest group became a protective factor for snus use in 2018, while being in the youngest group was a protective factor for smoking across all waves.

Overall, snus use development lends support to the hypothesis that the snus epidemic in Norway follows a similar development pattern as that of smoking and that the epidemic has entered stage III, according to the stages described by Lopez and colleagues [3, 4].

Despite similarities, the major difference between snus use and smoking development, in addition to prevalence, is the age distribution. In all waves, the oldest age group had the highest smoking prevalence, a finding consistent with the latter stage in the smoking epidemic [3, 4]. Regarding snus use, the findings are more mixed, but in the latter wave, snus use was most common in the oldest age group. This may be a result of a lower snus use initiation in young adults or a result of more late-onset snus use.

### Implications

If the snus use epidemic in Norway follows a similar pattern as that of smoking, and the snus epidemic is transitioning from stages II and III, then this will have several implications. First, the prevalence of male snus use has reached its peak and will not increase further. Second, female snus prevalence will increase until reaching the same or similar prevalence that male snus use had reached in 2018. Following the same trend from 2010 to 2018, female snus use will be similar to that of men in a few years. Third, snus use prevalence will start to decrease, first in men and then in women. Fourth, the social gradient in snus use will increase, similar to the social gradient in smoking.

The good news is that, from a public health perspective, if the snus use epidemic in Norway follows this predicted pattern, then snus use and tobacco use in general will begin to decline again. The change in snus user age distribution from 2010 to 2018 indicated that the decline may be imminent, as early onset snus use is dropping. Smoking has been steadily declining for decades [30], but the present study has shown that a rapid increase in snus use, especially among women, has brought the overall tobacco decline to a halt.

The study also found another emerging similarity between smoking and snus as young age became a protective factor for snus use in the latter wave, as it were for smoking in all waves. As early onset is considered a risk-factor for later use, a delayed onset may be seen as a protective factor.

If snus use begins to decline and there is not a new tobacco product rising proportionally, for example, e-cigarettes and the vaping of nicotine-containing liquids, which has increased substantially in the US in the last five years [31], general tobacco use will decline.

Regardless of a general decline or later onset in tobacco use, a switch from smoking to snus is good news both due to lower health hazards [23] and snus use not being found to be a gateway to smoking [25]. However, the latter findings are mixed [8]. Reports on whether other less harmful products such as e-cigarettes acts as gateways to smoking are also mixed [32–34], indicating a need for more research on whether less harmful products, including snus, may act as a gateway to smoking.

The bad news is, also from a public health perspective, the emerging social gradient in snus use, which combined with the health hazards associated with snus use will fuel social inequality in health. As mentioned, the health hazards with snus use are less severe compared to smoking [23], however snus use is still considered to be harmful. The present study lends support to the notion that snus use adds to social differences in health, as it shows a social gradient in snus use.

### Strengths and limitations

A major limitation in the study was the relatively modest participation rates in the surveys, ranging from 23% in 2010 to 31% in 2018 (detailed in Methods) [28, 29]. Such nonparticipation could be associated with key variables in this study, such as smoking, snus use, gender, age and socioeconomic status. This may lead to bias in the estimation of associations between these variables, and it may also reduce the study’s generalizability. In relation to this, the issue of sample comparability is important. As the surveys in 2014 and 2018 included somewhat different welfare organizations and institutions, a recent report using the same datasets, performed detailed sensitivity analyses of the HSCL-25, comprising only institutions that were included in all three surveys [28]. The results from these analyses showed near-identical effect-sizes of the trend data, suggesting that the three samples from 2010, 2014 and 2018 are comparable. Another limitation is that the present study is based on repeated cross-sectional data collection and not on a longitudinal study, which hinders the possibility of following individual and group trajectories. The study design also hinder the possibility to follow the social diffusion of snus use and smoking over time. The present study assumes that this social diffusions do not interact, which is considered a limitation. Further, the study only includes self-reported measures. The definition of low SES, which is only based on one question, is also considered a limitation. Further, the question is not commonly used, making comparing this study to similar study challenging. Another limitation is that the study is based on a survey including only people in higher education in Norway. However, as tobacco trends often start in higher SES classes, studying young people in higher education is especially important and may predict development in lower SES classes.

A major strength of the study is that it includes a large sample size in all three waves. In 2018, all full-time college and university students in Norway were invited to participate.

## Conclusions

The study, based on three large sample size waves from 2010 to 2018, supports a hypothesis that the snus epidemic follows a similar development in Norway as that of the smoking epidemic, as snus users are becoming more similar to smokers. This predicts that snus prevalence will not increase significantly more, at least not for men, and that its decline is imminent. However, it also suggests that snus use will be more prevalent in lower SES classes, a finding that this study also supports. Hence, snus use now seems to fuel the social gradient in health in a similar manner as that of smoking over recent decades.

## Data Availability

The SHoT dataset is administrated by the NIPH. Approval from a Norwegian regional committee for medical and health research ethics [https://helseforskning.etikkom.no] is a pre-requirement. Guidelines for access to SHoT data are found at [https://www.fhi.no/en/more/access-to-data].

## Abbreviation

SES: Socioeconomic Status
